# Covid-19 pandemic and its impact on increasing the risks of children's addiction to electronic games from a social work perspective

**DOI:** 10.1016/j.heliyon.2021.e08503

**Published:** 2021-12-01

**Authors:** Walaa Elsayed

**Affiliations:** College of Humanities and Science, Ajman University, Ajman, United Arab Emirates

**Keywords:** Electronic games, Addiction, Risks, Children, Social work

## Abstract

Children are among the social groups most affected by the COVID-19 pandemic because they have found themselves forced to stay at home, far from their schoolmates, their friends, and far from all the activities they used to do before the pandemic. so, it was their only refuge for recreation during their stay in Home is staying in front of the screens of tablets, smartphones, and computers to play electronic games for long hours, and there is no doubt that the sudden shift in the lifestyle of children during the Covid-19 pandemic had serious consequences and risks threatening their stability at all levels. In light of that, the current study aimed to determine the impact of the Covid-19 pandemic on increasing the social, psychological, behavioral, and health risks of children's addiction to electronic games from a social work perspective. This study falls under the type of descriptive-analytical studies that are based on describing the reality of the problem under study. The study sample included 289 children in the age group 6–17 years in the first grade to the twelfth grade at school. The researcher designed a questionnaire that reflects the four risks facing children to assess these risks. The results showed is that the value of all impacts of the Covid-19 pandemic on the increasing risks of children's addiction to electronic games came to a total weight of (27907), weighted relative weight of (80.47%). This indication is High, indicating that the level of impact is High for the Covid-19 pandemic on the increase in all types of risks of children's addiction to electronic games. It ranked first " Behavioral Risks " at 91.15%, It is followed by the ranked second “Social risks " at 85.5%, Then came third place " Psychological Risks" at 80.91%, and in finally in fourth place " Health Risks" at 64.28%, which necessitates the need to take a set of serious measures by educating parents to monitor the content of electronic games that their children play, especially violent games, in addition to, reduce the number of hours the child spends practicing these games, and to encourage parents to form a bridge of communication and constructive dialogue between them and their children, and that parents put controls and restrictions on their children's practice of electronic games to confront abnormal behavioral, psychological and social patterns such as aggression, violence, deception, lying, imitation, vigilance, physical stress, poor eyesight, distance from practicing religious rituals, academic delay, introversion, depression, intolerance, selfishness, sadness, isolation from society, social withdrawal and lack of forming social relationships and lack of communication with others. The researcher took care that the results of the current study are very accurate and representative of the reality of the research problem, in light of the researcher's emphasis on the commitment to observe ethical rules to ensure the confidentiality of data. finally, the current study will greatly benefit researchers interested in the field of childhood and its problems and they will rely on its results and recommendations in how to protect children from the dangers of electronic game addiction in light of the Covid-19 crisis in particular.

## 1 Introduction

The outbreak of the Coronavirus pandemic has led to repercussions that cast their shadows on all aspects of our lives, and no one was immune from its great effects that radically changed our way of life. Children are among the most affected social strata. They found themselves forced to stay home, away from their schools and classmates, and receive their education via the Internet (Daniel, 2020; Guan et al., 2020, Elsayed, 2021a, b, c, d). The pandemic restricted many of the recreational activities they used to, so the screens of tablets, smartphones, computers, and game consoles, such as the "PlayStation" and others, were their only refuge for recreation while they were staying at home. There was no escape for parents to reduce controls over the use of these devices, so that some families allowed their children to use the Internet throughout the day (Parker et al., 2021; Fischer and Haucap, 2020). There is no doubt that the sudden shift in the aforementioned lifestyle had dire consequences for children so that doctors and specialists notes changed in children's behavior and they began to receive more children suffering from behavioral problems, aggressive patterns, the decline in social skills, attention, and sleep disorders, which constituted a clear warning, and severe negative effects will increase in the case of developing into the stage of complete addiction, and then getting rid of it will be like getting rid of any other addiction, and its accompanying psychological and physical effects and symptoms such as anxiety, irritability, tremors, and nausea, Vision impairment and other serious symptoms that threaten the safety and security of children (Laato et al., 2020; Dunton et al., 2020; Cuffaro et al., 2020).

Since the beginning of the twentieth century, there has been great interest on the part of scientists and researchers in all fields with topics related to children's games because play occupies an important place in the child's life and it is considered one of the basic needs of the child, in addition to, it is the imaginary world that child lives with his toys and the way in which he expresses his happiness and sadness (Krahé and Möller, 2004; von Salisch et al., 2011). It is worth noting that playing has several benefits, including providing the child with the opportunity to express himself, liberation from restrictions, and strengthening the child's will and determination by being restricted to the system and foundations of the game, patience, endurance, waiting, and other developmental gains - physical, cognitive, emotional and social - in aspects of his personality (Tamborini and Skalski, 2006; Alexander et al., 2010). Therefore, the researchers interested by identifying the impact of games on children's behavior, perceptions, and lifestyle later. The interest appeared first within developed societies by giving the child more attention than the interest in the material economy because the children are the real investment in society, and the success of development plans depends on them, so the child from an early age is provided with a positive environment for linguistic, motor, emotional and mental development (Ferreira and Ribeiro, 2001; Parkes et al., 2013). Perhaps what distinguishes those early stages of the formation of the child's behavior is the interest in the materials that help him in play activities. Therefore, everyone and parents, in particular, must make sure to choose games that facilitate the mental development process of their children, because play is an important activity that the child practices, and it plays a major role in the formation of his personality on the one hand, and an emphasis on the value system of the society in which he lives sometimes, on the other hand, It is an educational and social activity that works to convert the incoming information to suit the needs of the child, and it is an innate activity through which the process of growth and development of the child's personality takes place (Arriaga et al., 2011; Straker et al., 2014).

Electronic games began to spread widely recently, as an American study by Gonigal (2011) showed that American children spend a long time playing electronic games equal to the time they spend in school learning. The phenomenon of electronic games addiction has become a global phenomenon that spreads among children and adolescents continuously, whether in homes or in Internet cafes (Straker et al., 2009, 2014; Przybylski, 2014).

Although there are some opinions that see that electronic games contribute to the development of the child in play, increase his skills, activate his areas of thinking, enrich his imagination and activate them in a wider direction, and push his abilities to growth and wide awareness, but at the same time they carry a lot of harm to the child, Especially on his physical, psychological, mental and behavioral health, and on the overall patterns of his culture in general, due to the negative and dangerous consequences of many electronic games, because a large proportion of electronic games depend on entertainment and enjoyment of killing others and destroying their property and assaulting them without right (Fromme, 2003; Olson et al., 2007). These games teach children and adolescents the methods of committing a crime, its arts, and tricks, and develop in their minds the abilities of bullying, aggression, and the consequences of the crime, These abilities are acquired through excessive persistence in playing these games, it is worth noting that these games may be more harmful than violent television films because they are characterized by an interactive character between them and the child, and they require the child to assume the aggressive character that he plays and practices in the electronic game, so many psychologists blame electronic games as being among one of the reasons that lead to the emergence of some behavioral disorders in the child and the problems of violence and bullying in the school environment, social isolation or introversion, in addition to some health problems, and the problem of poor academic achievement of the child (Wright et al., 2001; Papastergiou, 2009).

So children games, especially electronic games in our modern era are not a simple matter as many people imagine it, Game may reach a dangerous stage if it does not improve its choice, as it may have a bad or negative impact on the child's physical and psychological health when the child uses his game and practices it in a wrong way, or if it is the tools used in play are above the level of the child's age and mind. This needs attention on the part of adults to choose the appropriate games for their children so that they are appropriate for their age and level of thinking, in order to contribute to the development of the child's personality and behavior in a good manner (Maddison et al., 2007; Harrigan and Wardrip-Fruin, 2010).

### 1.1 The history of electronic games

The beginning of electronic games goes back to 1953 when some specialists were able to show a "louse" on a large screen of lamps and move it using a huge computer that cost millions of dollars at the time, followed by simplified simulations of games such as checkers and chess. In 1960, the space-war game designed by three students from MIT met with success that made the companies produce a valuable gift with the computer, and in the meantime, Ralf Baer designed the first home video game device called Magnavox Odyssey, which contained three ten games loaded with six bars (Dyson, 2017; Sabadello, 2006). The year 1972 witnessed a remarkable event in the history of electronic games, which is still developing and progressing steadily today. Nolan Bushnell and Ted Dabney founded an electronic games company in the United States of America, and they introduced the game "Pong", which soon met Unparalleled success, the game "Pong" was a simplified simulation of ping-pong, in which two tennis rackets were represented by two rectangles moving on both ends of the screen by two handles in the device, and a square ball moved between them, The general public flocked to this game when it was tested for the first time in a cafe, and the company was able in a short period of time to achieve great success by marketing more than one hundred thousand copies of this device (DeMaria, 2018). Faced with this success, Steve Jobs and Steve Wazniak rushed to introduce the game "Breakout", which is a wall consisting of pieces at the top of the screen that must be torn down with a ball and a horizontal racket located at bottom of the screen, this game has met with great demand and a huge success (Buckley and Anderson, 2006; Horne-Moyer et al., 2014).

There were many companies that began investing in developing and introducing various new games, thus achieving great profits that allowed them gradually to develop electronic devices and computers. Video games were no longer exclusively for certain layers of society, and their popularity was helped by the emergence of fun games that left an impact until today, such as the Pac-Man game (Yellow ball devouring targets and escaping hostile balls) and the popular Space Invaders game (Przybylski et al., 2014). The development and marketing of various devices and games accelerated, and in 1982 it reached its peak sales, then began the decline, which was contributed to a large extent by the emergence of gaming computers, until the sales of video games in 1985 became thirty times less than the sales in 1982, while Upon its advent in 1980, the personal computer could not compete with gaming devices due to the complex interactions that were in use at the time. And with the increasing need for devices that are easy to use and deal with, a Japanese company has launched a game machine with high-resolution graphic and graphic specifications, in which the idea of games depends on the adventures of a plumbing worker named Mario Mario who is looking for his princess (Cover, 2004). This device achieved widespread fame and fictional sales, until the sales of game cassettes on it exceeded fifty million tapes, prompting another Japanese company in 1968 to launch a similar product that relies on the character of a fast hedgehog named Sonic looking for his princess " the Hedgehog" The companies developed several games on their devices, achieving sales that exceeded all expectations, such as the Monaco Car Race and Zelda or Street Fighter. Personal computers developed in 1988, as processors 386 and 486 appeared, and sound pulses were soon replaced by sound cards, and the appearance of the Windows 3–11 program in 1990 allowed a simplified interaction with the computer that became accessible to many, so games became more fun and simulation games were called such as simulation Flying, simulating thinking games and chess games (Pergams and Zaradic, 2006).

In 1993, Pentium processors and CD-ROM readers appeared, and Microsoft introduced the Windows 95 program, which made the personal computer a powerful tool for developing multimedia games to benefit from images, graphics, and sounds, so its capabilities became greater and closer to reality. With the development of the personal computer, the decline of video game devices became so fast that some of them declared that the end of these devices is imminent, and large companies began to abandon one by one of their development programs in this field, but in 1995 a giant Japanese company introduced the Play Station gaming device, which is the provider (Al-Braizat, 2021). With a wide library of games with high capabilities in terms of sound, image, and speed, other Japanese companies soon followed suit, prompting the race to develop and market gaming hardware and software. Electronic games appeared independent of the computer or connected to it, controlled by the learner according to his orders within the artificial intelligence programs, and the robot (the human-machine), which imitated the work of man in his daily life, which gave these devices and games attached to them a wide variety of technologies, and allowed several senses to be occupied, such as sight, hearing and touching more proficiently (Esteves and Brito, 2020).

### 1.2 The concept of electronic games from a social work perspective

The electronic game is an activity in which players engage in an artificially disputed conflict that is governed by certain rules in a way that leads to quantifiable results, and it is called an electronic game if it is available in digital form, and it is usually played on computers, televisions, mobile phones and mobile devices such as laptops, iPad, PlayStation, Digital Versatile Disk (DVD) and virtual reality (Liu et al., 2020; Backe, 2020). Examples of electronic games include fun and excitement games that aim to entertain and occupy free time and depend on the user's interaction with the game and are distinguished by being exciting and drawing attention to the abundance of successive situations and the use of pictures and sound & visual stimuli, also, intelligence games that depend on logical simulation in making decisions (Wang et al., 2020a, b; Zyukina and Voropaeva, 2020).

Electronic games are defined as a recreational activity primarily mental and include all video games, computer games, and mobile phone games practiced by the individual individually or collectively through the Internet, and the electronic game is an organized game that has a base, goal, reactions, difficulty and a component of the challenge, competition and interaction, and the presentation method (Baptista and dos Reis, 2020; Ibrahim et al., 2020).

### 1.3 Reasons for the increased attraction of children to playing electronic games in light of the Covid-19 pandemic from a social work perspective

- It Features graphic, color, fantasy and adventure attractions (Barr and Copeland-Stewart, 2021).
- It depends on meditation and concentration, as electronic games require total focus, as well as science fiction (Pack and Hedlund, 2020).
- It depends on simulating heroes, as it relies on a hero who moves and changes his behavior according to the situations (Montag and Elhai, 2020).
- It Creates virtual imaginary worlds where the child lives in an imaginary world far from the truth, such as delusions of children that they are in fights in the jungle or invading space or prehistoric delusions such as fighting dinosaurs and others (Fontana, 2020).
- It relies on controlling and controlling events and people (Wunderlich et al., 2021).

### 1.4 The concept of electronic games addiction from a social work perspective

Electronic games addiction refers to the excessive or compulsive use of computer games or video games, which interferes with the daily life of a person, and the addiction to electronic games manifests itself in a sense of compulsion to play in a manner that results in social isolation, mood swings, diminished imagination, and an excessive focus on success in the game, all the way to cutting out other activities in life (Zubiaga and Cilleruelo, 2020; Abdallah, 2020; Kharisma et al., 2020).

### 1.5 Symptoms of electronic games addiction from a social work perspective

- Attachment to electronic devices.
- The child's unwillingness to play with other children.
- The child's unwillingness to go out in the open.
- Hold the child to stay in the places of video games.
- Changes in the child's temperament, stubbornness, convulsions and tension.
- Loss of focus in teens (Mukhtar, 2020; Venugopal et al., 2021).
- Obvious academic negligence.
- Creating arguments and excuses all the time.
- Find a source of funds to play in video gym clubs.
- Deceit and lie sometimes to get rid of the mother and father's questions.
- Loss of appetite and weight loss.
- Nervousness and excessive justification of situations (Wang et al., 2021; Singh and Paliwal, 2020).

### 1.6 Causes of electronic games addiction from a social work perspective

- Isolation (the condition created by the parents by lack of communication with the child).
- The leisure (the free time that a teenager can overcome by playing).
- Happiness (the euphoria that electronic games bring for children).
- Escape (the idea that the teenager lives to escape from reality).
- Professionalism (the tool that companies add to stimulation and addiction) (Afriwilda and Mulawarman, 2021; Mylona et al., 2020; Skoric et al., 2009; Brunborg et al., 2014; Chiu et al., 2004; Sahin et al., 2016).

### 1.7 Negative effects of electronic games addiction in light of the Covid-19 pandemic from a social work perspective

Although there are some opinions that see that electronic games contribute to the development of the child in playing, increase his skills, activate his areas of thinking, enrich his imagination and activate them in a wider direction, and push his abilities to growth and wide awareness, but at the same time they carry a lot of harm to the child, Especially on his physical, psychological, mental and behavioral health, and on the overall patterns of his culture in general, due to the negative effects and dangerous consequences of many electronic games (Boniel-Nissim et al., 2015; Fromme, 2003), as follows:

#### 1.7.1 Social risks

Addiction to electronic games destroys the child's social development by exposing him to the risk of introversion and social isolation, as the child loses social skills in dealing with others, is unable to speak, express and tact in speaking and becomes shy, in addition to, his suffering from social aggression that results from accompanying bad friends. Also, these games reduce the interaction between the child and his family, cause separation from reality and live in imagination, and negative cultivate values and concepts far from the traditions of community and religion values (Zamani et al., 2010; Griffiths, 1997; Elsayed, 2021a, b, c, d; Young and De Abreu, 2017).

It should be noted that the social isolation caused by electronic games is a manifestation of negative human behavior that has dangerous effects on the personality of the individual and his relationship with others, as it indicates his inability to engage in social relationships or to continue to engage in them, and to be confined or centered around himself, It may separate itself in this case from the selves of others, which indicates the insufficient attractiveness of the network of integrated social relations between the child and the individuals around him (Chiang et al., 2019; Gentile et al., 2011).

It can be said that social isolation and introversion are among the strong damages resulting from addiction to electronic games, because many of these games are designed in the way of solo play, which allows the child to distance himself from group play, and that unfortunately many families prefer and facilitate it because they see that it is better to isolate their child under the pretext of staying away from Problems and quarrels with other children, but this is not a solution, because the families that do that, they plan in this way intentionally or unintentionally to keep their children away from social educational situations in which the child learns the basics of dialogue and the pleasure of interaction and problem-solving, and thus the child loses the most important educational method, which is learning by trying And the error of experimentation and discovery through educational experimental situations, and therefore families that prefer this matter spoil the child more than they fix him, and this may be a cause of negative behaviors in socially isolated children (Gumport et al., 2021; Ray and Jat, 2010; Pontes, 2017).

So, addiction to electronic games takes a dangerous turn in its impact on the user in social images and forms, which makes the user live in a missing link away from communicating with others, which would lead to social isolation and introversion of the child addicted to these games. As many specialists believe that the risk of children and adolescents being isolated in their inability to express the condition they are afflicted with, which makes the isolated individual feel overly sensitive in social interaction due to his lack of experience in life, as well as that he frequently resort to disordered behaviors and behaviors, such as desire In suicide, escaping from school, or resorting to aggression in behavior, given that a person in his teens is intellectually and psychologically disturbed, and therefore has the willingness to practice aggressive behaviors towards himself and others, and this has been confirmed by many studies that indicated that there is An increase in the rate of aggression among socially isolated children and adolescents compared to their non-isolated peers (Chou and Ting, 2003; Lam et al., 2009; Leung, 2014).

#### 1.7.2 Psychological risks

Addiction to electronic games destroys the psychological development of the child by exposing him to feelings of fear, tension, distress and insomnia due to the terrifying and negative contents of some electronic games, as well as the child's exposure to withdrawal anxiety disorders such as excessive anxiety, depression and severe sadness, in addition to developing the desire for violence and cruelty in the child (Wan and Chiou, 2006; Elsayed, 2021a, b, c, d; Lissak, 2018). Specialists in the field of childhood indicated that among the most prominent psychological and emotional damages facing children who overuse electronic games such as depression, sleep disorders, violence and aggression, disease phobia, neglect of communication, schizophrenia, drug and alcohol abuse and suicide (many cases were suicides due to the blue whale game as an example) (Whang et al., 2003; Beard and Wolf, 2001).

It also Addiction to electronic games destroys the emotional development of the child, and the child becomes exaggerated/indifferent in his reactions, and he has nervousness, over-excitement and an exaggerated reaction, in addition to immaturity disorders such as the ability to not focus and distracted thinking, lack of tolerance, impatience, laziness, neglect, lethargy and lack of independence, in addition to The mood of the child is aggressive, and it is characterized by agitation and responses that are volatile and unstable (King et al., 2020; Mehroof and Griffiths, 2010).

#### 1.7.3 Behavioral risks

The addiction to electronic games leads to the emergence of aggressive behaviors such as violence and bullying in children and adolescents, so they have what is called physiological agitation that leads to automatic violence, rebellion, lethargy and laziness, not hearing instructions and directions by their parents, and their injury to behavioral and emotional disorders that represent the abnormal behavior represented in violence that It learns from electronic games, children who play violent games show a tendency to behave aggressively in reality, as these games promote violence, crime and bad morals such as learning of fraud which affects the behavior of children and their interaction with others negatively (Ko et al., 2007; Setzer and Duckett, 1994; Bingham et al., 2013). It also works to spread the negative culture represented by the culture of aggressive conflicts that these games carry in their various forms and contents, those many conflicts that threaten the positive culture and work to undermine the positive values of the child and negatively affect the child's being and his disciplined behaviors, to be replaced by the culture of violence and its tendency to devote Its values and presence in the child's behavior and culture, and this leads to high crime rates due to these behaviors such as crimes (murders and serious attacks on public and private property in many societies) (Grüsser et al., 2006; Elsayed, 2020; Peng and Liu, 2009).

Many psychological researchers have found that most of the games used by children and adolescents contain negative connotations, which may affect them in all stages of their development, and also generate some unwanted behaviors such as bullying behavior, especially since these electronic games tend mostly to the side of violence and conflict between two teams or between two players, and embodied in the mentality of the son that life is all a fight, and he forgets the principles of dialogue, understanding, cooperation and integration (Dahabiyeh et al., 2020; Muhamad and Kim, 2020). It should also be noted that children are attracted to imitating their favorite heroes in electronic games, and this is what makes them assume their personalities according to the principles and values of the hero they prefer, and this in turn affects the formation of their characters and leads them to practice violence through real situations in life (Mohammed and Ali, 2020; Yang et al., 2020).

#### 1.7.4 Health risks

Addiction to electronic games exposes the child to stress and poor eyesight in the long run as a result of sitting for long periods in front of the electronic screen, and also leads to obesity and brain disorders, especially concentration and thinking disorders, so the child becomes apathetic (Mundy et al., 2020; Chen et al., 2020), in addition to feeling headache and physical, muscle and bone stress, as well as the child's exposure to back and hand problems which may lead to health risks and injuries may end with disabilities such as injuries to the limbs, neck and back when sitting in a wrong way in front of these games for long periods as a result of the child or teenager not doing any simple exercises during sitting time, as well as severe damage to the thumb due to the large movement of the fingers during play and bending it continuously, in addition to the intermittent, contrasting flash of lighting in the animation may lead to seizures of epilepsy in the child, and the increased use of vibrating computer games also causes the disease of tremor of the arms, and health experts confirmed that the child's eyes are also negatively affected when playing for long periods of these games, as the movement of the eyes is rapid, which leads to stress and the occurrence of redness, dehydration and dizziness due to the electromagnetic radiation emitted from the electronic screen, and then the child begins to feel a headache and sometimes depression (Wang et al., 2020a, b; BAHRİLLİ et al., 2020; Han et al., 2020).

Health professionals also emphasized that among the most important physical damages that occur to the child, such as lower back, backbones pain, neck pain, muscle sluggishness, poor vision, weak bladder muscles, loss of appetite, weight loss and chronic constipation (McLeod et al., 2017; Elsayed, 2021a, b, c, d; Barbero et al., 2018; Das et al., 2017).

### 1.8 The most electronic games that pose a serious threat to children's lives from a social work perspective (5 games that cause murder and suicide among children)

- **Counter Strike game:** It is the game that caused more accidents than others due to the player's inability to differentiate between real reality and virtual reality, where one teenager killed a person who killed him inside the game, while another teenager killed his grandmother and injured his grandfather because he did that in the game (Makarov et al., 2017; Hellerstedt and Mozelius, 2018).
- **Grand Theft Auto game:** In this game also during which the player loses the distinction between reality and fiction, for example two teenage friends used a machine gun to snipe motorbikes because it is the same as what they do in the game, and another teenager retired to surprise a person passing by his rifle towards him to kill him quickly because he believed that this is how he would win in the game (Coyne and Stockdale, 2021; Gabbiadini et al., 2017).
- **Blue whale game:** Although the Blue Whale game does not make children and adolescents difficult to separate between fact and fiction, it asks them to do strange and illogical actions to reach the next levels, and in the last five levels it asks them to carry out acts of violence and kill others or kill themselves, as this game causes cases Deaths and suicides exceeded a hundred in many different countries (Mukhra et al., 2019; Khattar et al., 2018).
- **Mariam game:** It is a game that relies heavily on frightening children and adolescents, as it is considered the first horror game in the world, and focuses on the child's loss of consciousness due to terror, and the game asks for any requests from the child to return to his normal life, and from those requests to carry out violent behaviors against himself or others, but some children did not tolerate the idea of constant terror and afraid, so they committed suicide (Jean, 2019; Al-Dhlan and Al-reshidi, 2021).
- **Momo game:** It is a game that teenagers play through WhatsApp, and it can completely control children, then force them to do the actions required to get rid of "Momo" that watches them all the time, and suicides have been recorded due to the Momo game, which some Internet experts accused of being games aimed at penetration smart phones (Kobilke and Markiewitz, 2021; Mortada Fouda and Ali Mohamed Abdel-Rahman, 2020).

### 1.9 Stages of influence of the child with electronic games from a social work perspective

- **Imitation:** In this stage, the child assumes the hero character who loves his behavior, and he wishes to behave with his characteristics (Lobel et al., 2017; Campbell and Cunnington, 2017).
- **Saturation and acquisition:** In this stage, the child acquires all the attributes and behaviors that are consistent with what he wants and follows them continuously and routinely without awareness (Pitt et al., 2017; Li et al., 2019).
- **The absence of the human conscience:** In this stage, the child acquires characterized by the absence of the inner prohibitions in terms of activity and expression, and here the child moves to the stage of action (Slater et al., 2017; Sarkar, 2019).
- **Sagging individual sense:** At this stage, the child adapts to violent events as a result of their recurrence, so he is no longer affected by them and is seen as nature and it is considered to him a way of life (Mazur et al., 2018; Baldwin, 2019).

### 1.10 The justification for this study, and the new significance behind it (importance of study)

- This study will show that children in light of the Covid-19 pandemic are among the most negatively affected groups due to the long period of their stay at home, because they spend most of their time in front of electronic devices to play electronic games, especially violent ones, and this is a dangerous indicator that threatens the stability and safety of the child.
- This study will clarify that the phenomenon of Covid-19 is a new phenomenon in the whole world and has real effects on children, and therefore this study will clarify those effects specifically about the impact of this pandemic on increasing children's accustomed and addiction to using electronic games for a long period of time
- This study will highlight the nature of the social, health, psychological and behavioral risks resulting from children's addiction to electronic games and the extent of the impact of these problems on their stability within their families from a social work perspective.
- This study will clarify the child's right to be protected from every danger he is exposed to when excessive use of electronic games in light of the Covid-19 pandemic by providing serious recommendations from a social work perspective to work to mitigate these risks during crises generally and the Covid-19 pandemic crisis especially

### 1.11 The impact of the current study in the United Arab Emirates

The data collection of the study sample in the United Arab Emirates had a very important impact, this effect represented in standing on the actual reality of the phenomenon of children excessive addiction to the use of electronic games during the crisis of the Covid-19 pandemic, in order to educate parents in the United Arab Emirates on the need to monitor their children to protect them from the dangers of addiction to electronic games during this covid-19 crisis, with working to integrate their children into the practice of various household activities as a means of entertainment until this crisis ends and life returns to normal.

### 1.12 The present study and it questions

The present study is concerned with studying the impact of the Covid-19 pandemic on increasing risks of children's addiction to electronic games from a social work perspective, and this is a new aspect not addressed before. In order to do so, this research asks the following research questions:

***Main question of the study:*** What is the impact of the Covid-19 pandemic on increasing risks of children's addiction to electronic games from a social work perspective?

This main question of the study will be answered by answering the following sub-questions:

**Q1:** What is the impact of the Covid-19 pandemic on increasing Social risks of children's addiction to electronic games from a social work perspective?
**Q2:** What is the impact of the Covid-19 pandemic on increasing Psychological risks of children's addiction to electronic games from a social work perspective?
**Q3:** What is the impact of the Covid-19 pandemic on increasing Behavioral risks of children's addiction to electronic games from a social work perspective?
**Q4:** What is the impact of the Covid-19 pandemic on increasing Health risks of children's addiction to electronic games from a social work perspective?
**Q5:** Does the degree of children's awareness of their increased exposure to the risks of addiction to electronic games due to the Covid-19 pandemic vary according to gender, age, number of hours of play per day, and favorite games?

## 2 Methodology and procedures

### 2.1 Sample

The study community is represented by all children in the age group 6–17 years in the first grade to the twelfth grade at school with an approximate total of (12197) children from the United Arab Emirates. The study sample was selected at random, the sample size was approximate (289) children from the United Arab Emirates, and the researcher used a simple random survey (a representative sample of the study population of abused children). To calculate and measure a sample size, the researcher used Cochran's Equation as shown in Eq. (1) (Cochran, 1963):

(1) $$n=\frac{n_{0}}{1+\frac{\left(n_{0}-1\right)}{N}}$$

The sample size in limited populations, which refers to the study population, is denoted by **n**, the sample size in infinite communities is denoted by **n**_**0**_ (open communities), and the size of the study population is denotes **N**, where the study population was identified using official data from The Ministry of Education in the United Arab Emirates, which was calculated from reality of the records is 12197 children, and the researcher calculated a **n**_**0**_ using Smith's Equation as shown in Eq. (2) (Smith, 1983)

(2) $$n_{0}=\frac{Z^{2}\sigma^{2}}{e^{2}}$$

where n_0_ is the sample size, z is the abscissa of the normal curve that cuts off an area α at the tails and the researcher determined it by 99% at the level of significance of 1%, which is estimated at ± 2.58., e is the desired level of precision (in the same unit of measure as the variance) which was determined by the researcher as only one degree, and σ is the variance of an attribute in the population.

By doing the calculations it was→:

$${\text{n}}_{0}=\frac{{\left(2.58\right)}^{2}\times {\left(6.67\right)}^{2}}{{\left(1\right)}^{2}}=296$$

The following formula can be used to measure the sample size in the research population

$$\text{n}=\frac{{\text{n}}_{0}}{1+\frac{\left({\text{n}}_{0}-1\right)}{\text{N}}}=\frac{296}{1+\frac{\left(296-1\right)}{12197}}=289\;\text{Children}$$

In the Current research, the sample consisted of 289 children (see Table 1 shows the demographic information on participants).

**Table 1:** Demographic information on participants.

Variables	Statement	Frequencies	Percentage
Gender	Male	187	64.7%
	Female	102	35.3%
	**Total**	**289**	100%
Age	6–8 years	65	22.5%
	9–11 years	83	28.7%
	12–14 years	79	27.3%
	15–17 years	62	21.5%
	**Total**	**289**	100%
Number of hours of play per day	1 h	40	13.9%
	2 h	57	19.7%
	3 h	46	15.9%
	4 h	61	21.1%
	5 h or more	85	29.4%
	**Total**	**289**	100%
Favorite electronic games	Simulation games	31	10.7%
	Adventure games	18	6.2%
	Puzzle Games	27	9.3%
	Action games	42	14.5%
	Shooting and stealth games	42	14.5%
	Fighting Games	66	22.9%
	Sports games	34	11.8%
	Role-playing games, character and story	21	7.3%
	Educational games	8	2.8%
	**Total**	**289**	100%

In light of these results, it is clear that the percentage of male represents the highest rate at 64.7%, and followed female at 35.3%. Also, the majority of children are those in the age group of 9–11 years at 28.7%, followed by 12–14 years at 27.3%, followed by 6–8 years at 22.5% and followed by 15–17 years at 21.5%. Besides, it is clear that the majority of children spend big Number of hours of play per day from 5 h or more at 29.4%, followed by 4 h at 21.1%, followed by 2 h at 19.7%, then by 3 h at 15.9%, and followed by spend 1 h at 13.9%. In addition to, it turns out that the most popular electronic games children prefer to play are fighting games at 22.9%, followed by action game, shooting and stealth games at 14.5%, followed by sports games at 11.8%, followed by Simulation games at 10.7%, followed by puzzle games at 9.3%, followed by role-playing games, character and story at 7.3%, followed by adventure games at 6.2%, then educational games at 2.8%.

### 2.2 Ethical approval

Before starting the survey, all children participants and their parents gave their informed consent to apply the Questionnaire to their children. Participants' data were completely analyzed without revealing their identity. The approval of the Scientific Research Ethics Committee in UAE, has been obtained. In addition to assurance that the researcher followed all applicable ethical regulations to keep the participants' details confidential.

### 2.3 Research method

Since this study falls under the descriptive research pattern, which seeks to explain and evaluate the variables of the study so that the researcher can obtain reliable data and information to represent the reality of the situation, the researcher used descriptive analysis to collect, analyze, and interpret the data for the study methodology. Descriptive analysis is a method of a systematic and analytical approach to describe and quantify phenomena to arrive at accurate results through the explanation, comparison, and interpretation, as to the nature of risks of children's addiction to electronic games, in addition to determining the differences between the degree of awareness of children to the risks of their addiction to electronic games in light of the Covid-19 pandemic according to gender, age, number of hours of play per day, and favorite games. This is in light of the monitoring, analysis, and interpretation of the data that was accessible from the study sample, extracting accurate conclusions and recommendations.

### 2.4 Study instrument and design the survey

**Questionnaire:** The researcher designed a questionnaire on the risks of child addiction to electronic games in terms of risks (social - psychological - health - behavioral) due the Covid-19 pandemic from a social work perspective. This questionnaire was prepared by referring to the theoretical framework to the Current study, and referring to the previous studies related to the current study in order to determine the dimensions of the study, then the researcher identified and formulated the phrases for each dimension, and the researcher managed the validity and reliability of the questionnaire as follows:

- Validity of the questionnaire**:** The research tool was confirmed by the virtual validity method for the questionnaire by presenting it in its initial form with a list of study questions, to 17 members of the teaching staff of universities to arbitrate the questionnaire, and the content of questionnaire was adjusted according to their recommendations, and an agreement percentage of not less than 85% was relied on, so some phrases were deleted and others phrases were reformulated, and accordingly the questionnaire was formulated in its final image according to the recommendations of the arbitrators' opinions. The final number of the questionnaire's questions was 40 questions distributed over the four dimensions of the questionnaire (10 questions for each dimension in the questionnaire).
- Reliability of the questionnaire: The researcher verified the reliability of the questionnaire by using the test-retest method. The questionnaire was applied to a small random sample consisting of 40 children, and fifteen days after, the test was reapplied to the same sample of children again, after that, the Spearman correlation coefficient between the two applications was calculated, it is worth noting that the reliability coefficient was calculated according to Spearman's law of correlation coefficient as shown in Eq. (3): -

(3) $${\text{R}}_{\text{s}}=1-\frac{6\left(\sum {\text{D}}^{2}\right)}{\text{n}\left({\text{n}}^{2}-1\right)}$$

In light of results in Table 2., the correlation coefficients of the Questionnaire and its axes are high, which makes us confident in the stability of the resolution, and the self-validity factor was calculated by calculating the square root of the stability factor of the resolution, and the self-validity of the resolution was = 0.76 = 87%, Therefore, the questionnaire form is characterized by validity and stability, given that all statements of the tool's axes have a high degree of validity and reliability.

**Table 2:** Stability of the questionnaire and its variables.

Variables of the questionnaire	Reliability (Rs)	Validity (Vs)
Social risks of children's addiction to electronic games	0.77	.88
Psychological risks of children's addiction to electronic games	0.79	.89
Health risks of children's addiction to electronic games	0.73	.85
Behavioral risks of children's addiction to electronic games	0.75	.86
**TOTAL**	0.76	.87

### 2.5 Data collection and analysis measures

⁃ The data was collected after creating an electronic questionnaire on the Google Form platform and applying it to 289 children who use electronic games continuously, and the period taken to collect the data was 6 months from November 2020 to April 2021,
⁃ The children in UAE answered all the survey questions to find out their views about the degree of the risks of their Addiction to Electronic Games due to the Covid-19 pandemic.
⁃ The researcher relied on a three-dimensional Likert scale {Agree (3), Neutral (2) Disagree (1)}, which determined the degree to the participants responded to each statement of the questionnaire. The justification for the researcher's use of the 3-point Likert scale is because the 3-point Likert scale provides freedom for respondents in the quality of the “feedback” observations they provide, where the question is asked to find out the participants' opinion on a particular topic or the degree of respondents' agreement with a particular statement, and the answer is usually from a 3-point Likert scale "agree and disagree as polarity points", in addition to “neutral choice”, then each opinion is given its weight (Weights). Also, this scale is also suitable for the type and size of the sample and the nature of the items and sentences included in the questionnaire to gain insight into the feelings, opinions, impressions, and behaviors of the respondents about a set of items that surround the phenomenon or problematic situation being investigated (Taherdoost, 2019; Flaskerud, 2012; Joshi et al., 2015).
⁃ I would like to clarify that children at the age of 6 years were able to answer the survey question themselves because they have completed kindergarten and have the ability to read, in addition to the vast majority of children starting at the age of 4 years in the UAE, they get used to using electronic devices significantly, and this enabled them to develop their abilities in reading and writing online on electronic devices. It is worth mentioning, Many scientific studies have proven that the brain of children at the age of 3–5 years absorbs a huge amount of new words that are engraved in their memory, and their linguistic growth rate may reach to accept 900–2500 new words, and sentences become longer, more quality and complex (Look: Altinkamis and Simon, 2020; Thorpe, 2006) by accustoming parents to their children on reading and writing continuously, and this is what develops their language abilities through questions and answers and learning in fields of knowledge, whether in art or science or even while playing and practicing sports activities (Look: MacLeod et al., 2020; Jasińska et al., 2020). It should also be noted that the first group in the age group of 6–8 years in the current study includes children who have become in a new educational stage after the kindergarten stage, which is the primary stage, and therefore this age is able to reading, and answering the questions asked.
⁃ And since the questionnaire consisted of 40 statements on four axes, and the number of the study sample members are 289 children, So, to calculate the one-axis level of addiction to electronic games, the range will be calculated by determining the difference between the highest and lowest value that can be obtained as follows:

Highest value: 289 × 10 × 3 = 8670.

Minimum value: 289 × 10 × 1 = 2890.

So the range = 8670–2890 = 5780.

Thus, 5780/3 = 1926.

Accordingly, the levels of one axis of addiction to electronic games will be divided as follows:

- Low addiction axis level (2890–4816)
- Middle addiction axis level (4817–6743)
- High addiction axis level (6744–8670)

So, to calculate the all levels of addiction to electronic games questionnaire with its four axes, the range will be calculated by determining the difference between the highest and lowest value that can be obtained as follows:

Highest value: 289 × 40 × 3 = 34680.

Minimum value: 289 × 40 × 1 = 11560.

So the range = 34680–11560 = 23120.

Thus, 23120/3 = 7706.

Accordingly, the levels of all levels of addiction to electronic games questionnaire with its four axes will be divided as follows.

- Low addiction level (11560–19266)
- Middle addiction level (19267–26973)
- High addiction level (26974–34680)

### 2.6 Statistical analysis

To analyze and interpret the data, the researcher used the Statistical Package for the Social Sciences (SPSS) analytical tools, as well as some statistical coefficients to address the study questions, which were (frequencies, percentages, weighted relative weights, total weights), In addition to the one-way ANOVA test, which is used to determine the importance of variations in averages.

## 3 Results

### 3.1 Study findings related to RQ1

The question was: What is the impact of the Covid-19 pandemic on increasing social risks of children's addiction to electronic games from a social work perspective?

The social risks of children's addiction to electronic games due Covid-19 pandemic have been identified by using the Questionnaire instrument and then arranging these social risks according to their total weights, weighted relative weight, and percentage from High to low (see Table 3).

**Table 3:** The social risks of children's addiction to electronic games due Covid-19 pandemic (N = 289).

The social risks of children's addiction to electronic games due Covid-19 pandemic	Total weights	Weighted relative weight %	percentage %	Ranking
I fell into many disputes and problems with my parents and sisters as a result of my excessive preoccupation with electronic games	746	86.04	10.06	4
I became less sitting and interacting with my family members due to my great interest in electronic games	804	92.73	10.84	1
I became uninterested in performing my social roles due to my intense love for electronic games	730	84.2	9.84	5
The social relations between me and my family members deteriorated due to my excessive interest in electronic games	774	89.27	10.44	2
Interacting with my classmates and participating with them in school activities became very little due to my preoccupation with electronic games	728	83.97	9.82	6
I tend to not attend social events of my family, colleagues, and friends because of my preoccupation with electronic games	772	89.04	10.41	3
I lost a lot of my friends because I was busy spending my free time playing electronic games rather than talking to them	725	83.62	9.78	7
I became less talkative and lost tact in speaking as a result of my merging with electronic games	713	82.24	9.61	9
My ability to express what was inside me in different situations became weak due to my constant preoccupation with electronic games	717	82.7	9.67	8
The advice of my parents and expressing their opinions about my life and school matters has become very little as a result of my merging with electronic games.	707	81.55	9.53	10
Total	7416		100%	

Weighted relative weight of the variable	85.5%
Level of weight representation	High

Table 3. We note from the results that the impact of the Covid-19 pandemic on the increase the social risks of children's addiction to electronic games were obtained a total weight of (7416), this is located between the High addiction axis level (6744–8670), So this indication is High, indicating that the level of impact is High. It is clear from the analysis that the phrase (I became less sitting and interacting with my family members due to my great interest in electronic games) showed the highest percentage of the level of Social risks of electronic games addiction, with total weights (804), Weighted relative weight (92.73), percentage (10.84), and Ranking (1). Then the phrase (The social relations between me and my family members deteriorated due to my excessive interest in electronic games), with total weights (774), Weighted relative weight (89.27), percentage (10.44), and Ranking (2). Then the phrase (I tend to not attend social events of my family, colleagues, and friends because of my preoccupation with electronic games), with total weights (772), Weighted relative weight (89.04), percentage (10.41), and Ranking (3). Then the phrase (I fell into many disputes and problems with my parents and sisters as a result of my excessive preoccupation with electronic games), with total weights (746), Weighted relative weight (86.04), percentage (10.06), and Ranking (4). Then the phrase (I became uninterested in performing my social roles due to my intense love for electronic games), with total weights (730), Weighted relative weight (84.2), percentage (9.84), and Ranking (5). The 5 types of Social risks of electronic games addiction that we have reviewed are the most types of social risks that negatively affect the child, which has clearly increased after the Covid-19 pandemic, this threatens the child with great danger towards this increasing rate of Social risks directed against him, which affects his normal relations with his family members. This corresponds to a study (Pontes, 2017; Cha and Seo, 2018; King et al., 2019; Gong et al., 2019; and Balakrishnan and Griffiths, 2018) which emphasized Addiction to electronic games destroys the child's social development by exposing him to the risk of loses social skills in dealing with others, is unable to speak, express and tact in speaking and the child becomes shy, in addition to, his suffering from social aggression that results from accompanying bad friends.

### 3.2 Study findings related to RQ2

The question was: What is the impact of the Covid-19 pandemic on increasing Psychological risks of children's addiction to electronic games from a social work perspective?

The Psychological risks of children's addiction to electronic games due Covid-19 pandemic have been identified by using the Questionnaire instrument and then arranging these psychological risks according to their total weights, weighted relative weight, and percentage from High to low (see Table 4).

**Table 4:** The Psychological risks of children's addiction to electronic games due Covid-19 pandemic (N = 289).

The Psychological risks of children's addiction to electronic games due Covid-19 pandemic	Total weights	Weighted relative weight %	percentage %	Ranking
I prefer to isolate myself from others to spend more time with electronic games	761	87.77	10.85	3
I feel very frustrated when I lost in electronic games	784	90.43	11.18	1
I love my imaginary world that electronic games give me more than the real world I live in	766	88.35	10.92	2
I feel very jittery and bad mood when someone forbids me from playing electronic games	745	85.93	10.62	4
I get angry to my siblings when their opinions interfere with my choices about the nature of the electronic games that I play	679	75.7	9.68	7
I feel lazy, lethargic, and inactive due to the frequent practice of electronic games	612	70.59	8.72	10
I see that my love for myself more than others is an advantage that I learned from the large number of electronic games I practice	614	70.82	8.75	9
I see that my dependence on those around me gives me more time to play electronic games	713	82.24	10.16	5
My attraction and love for electronic games is increasing day by day to the point that I cannot do without them at all.	683	78.78	9.74	6
I hate anyone who thinks they are better than me in playing electronic games	658	75.9	9.38	8
Total	7015		100%	

Weighted relative weight of the variable	80.91%
Level of weight representation	High

Table 4. We note from the results that the impact of the Covid-19 pandemic on the increase The Psychological risks of children's addiction to electronic games were obtained a total weight of (7015), this is located between the High addiction axis level (6744–8670), So this indication is High, indicating that the level of impact is High. It is clear from the analysis that the phrase (I feel very frustrated when I lost in electronic games) showed the highest percentage of the level of Psychological risks of electronic games addiction, with total weights (784), Weighted relative weight (90.43), percentage (11.18), and Ranking (1). Then the phrase (I love my imaginary world that electronic games give me more than the real world I live in), with total weights (766), Weighted relative weight (88.35), percentage (10.92), and Ranking (2). Then the phrase (I prefer to isolate myself from others to spend more time with electronic games), with total weights (761), Weighted relative weight (87.77), percentage (10.85), and Ranking (3). Then the phrase (I feel very jittery and bad mood when someone forbids me from playing electronic games), with total weights (745), Weighted relative weight (85.93), percentage (10.62), and Ranking (4). Then the phrase (I see that my dependence on those around me gives me more time to play electronic games), with total weights (713), Weighted relative weight (82.24), percentage (10.16), and Ranking (5). The 5 types of Psychological risks of electronic games addiction that we have reviewed are the most types of Psychological risks that negatively affect the child, which has clearly increased after the Covid-19 pandemic, this threatens the child with great danger towards this increasing rate of Psychological risks directed against him, which affects his normal relations with his family members. This corresponds to a study (Saquib et al., 2017; Wang et al., 2019; Chiang et al., 2019; Hing et al., 2017; and Novrialdy et al., 2019) which emphasized that Addiction to electronic games destroys the psychological development of the child by exposing him to feelings of fear, tension, distress, excessive anxiety, depression, insomnia, and severe sadness in the child due to the terrifying and negative contents of some electronic games.

### 3.3 Study findings related to RQ3

The question was: What is the impact of the Covid-19 pandemic on increasing Behavioral risks of children's addiction to electronic games from a social work perspective?

The Behavioral risks of children's addiction to electronic games due Covid-19 pandemic have been identified by using the Questionnaire instrument and then arranging these Behavioral risks according to their total weights, weighted relative weight, and percentage from High to low (see Table 5).

**Table 5:** The Behavioral risks of children's addiction to electronic games due Covid-19 pandemic (N = 289).

The behavioral risks of children's addiction to electronic games due Covid-19 pandemic	Total weights	Weighted relative weight %	percentage %	Ranking
I see violence against others as a manifestation of strength that I learned from electronic games	822	94.81	10.4	2
My electronic games taught me the use of weapons, especially in combat games	815	94	10.31	3
I mimic the behaviors and actions I see in the electronic game	796	91.81	10.07	5
I am convinced of electronic games content that assaulting others is justifiable in some situations	808	93.19	10.22	4
I learned from electronic games content that deception and lies are permissible, to I win on my opponent in front of me	792	91.35	10.02	6
I spend all my personal money on buying new electronic games	755	87.08	9.56	9
My religious performance decreased a lot after playing electronic games	743	85.7	9.4	10
I criticize everyone around me for not seeing persons as heroes like the ones I see in my electronic games.	775	89.39	9.81	7
I hit my sisters and friends in the same way that I see in electronic games	830	95.73	10.5	1
My role models and my ideals in life are the heroes of electronic games because I adopt their same convictions	767	88.47	9.71	8
Total	7903		100%	

Weighted relative weight of the variable	91.15 %
Level of weight representation	High

Table 5. We note from the results that the impact of the Covid-19 pandemic on the increase the behavioral risks of children's addiction to electronic games were obtained a total weight of (7903), this is located between the High addiction axis level (6744–8670), So this indication is High, indicating that the level of impact is High. It is clear from the analysis that the phrase (I hit my sisters and friends in the same way that I see in electronic games) showed the highest percentage of the level of behavioral risks of electronic games addiction, with total weights (830), Weighted relative weight (95.73), percentage (10.5), and Ranking (1). Then the phrase (I see violence against others as a manifestation of strength that I learned from electronic games), with total weights (822), Weighted relative weight (94.81), percentage (10.4), and Ranking (2). Then the phrase (My electronic games taught me the use of weapons, especially in combat games), with total weights (815), Weighted relative weight (94), percentage (10.31), and Ranking (3). Then the phrase (I am convinced of electronic games content that assaulting others is justifiable in some situations), with total weights (808), Weighted relative weight (93.19), percentage (10.22), and Ranking (4). Then the phrase (I mimic the behaviors and actions I see in the electronic game), with total weights (796), Weighted relative weight (91.81), percentage (10.07), and Ranking (5). The 5 types of behavioral risks of electronic games addiction that we have reviewed are the most types of behavioral risks that negatively affect the child, which has clearly increased after the Covid-19 pandemic, this threatens the child with great danger towards this increasing rate of behavioral risks directed against him, which affects his normal relations with his family members. This corresponds to a study (Karlsson et al., 2019; Männikkö et al., 2020; and Nogueira et al., 2019) which emphasized that addiction to electronic games leads to the emergence of aggressive behaviors such as violence and bullying in children, so they have what is called physiological agitation that leads to automatic violence, rebellion, lethargy, and laziness, not hearing instructions and directions from their parents.

### 3.4 Study findings related to RQ4

The question was: What is the impact of the Covid-19 pandemic on increasing Health risks of children's addiction to electronic games from a social work perspective?

The Health risks of children's addiction to electronic games due Covid-19 pandemic have been identified by using the Questionnaire instrument and then arranging these Health risks according to their total weights, weighted relative weight, and percentage from High to low (see Table 6).

**Table 6:** The Health risks of children's addiction to electronic games due Covid-19 pandemic (N = 289).

The Health risks of children's addiction to electronic games due Covid-19 pandemic	Total weights	Weighted relative weight %	percentage %	Ranking
I developed visual impairment and eye inflammation as a result of the long time I was sitting in front of the electronic game screen	577	66.55	10.35	3
My weight increased significantly, and I became obese due to my lack of movement to my preoccupation with electronic games.	622	71.74	11.16	1
I became ill with depression as a result of the intense fear that I was exposed to during a game	562	64.82	10.08	5
I have permanent insomnia and sleep disturbances as a result. I am constantly exposed to radiation emanating from computers or smartphones when playing games.	535	61.71	9.6	7
I see that playing with electronic games causes distraction of my attention, concentration for long periods of time, which has negatively affected academic achievement.	530	61.13	9.51	9
I suffer from migraine as a result of playing with electronic games a lot.	516	59.52	9.26	10
I suffer from severe pain in the shoulders and neck and inflammation in the spine due to the lack of movement while playing electronic games	585	67.47	10.5	2
I suffer from a tear in the ligaments of the joints of the hands as a result of excessive movement of my fingers on the arm of electronic games	564	65.05	10.12	4
I suffer from vitamin D deficiency as a result of not being exposed to the sun constantly due to the lack of going out of the house	533	61.48	9.57	8
I feel dizzy as a result of my lack of sleep and my disinterest in eating food due to my preoccupation with electronic games	549	63.32	9.85	6
Total	5573		100%	

Weighted relative weight of the variable	64.28%
Level of weight representation	Middle

Table 6. We note from the results that the impact of the Covid-19 pandemic on the increase the health risks of children's addiction to electronic games were obtained a total weight of (5573), this is located between the Middle addiction axis level (4817–6743), So this indication is Middle, indicating that the level of impact is Middle. It is clear from the analysis that the phrase (My weight increased significantly, and I became obese due to my lack of movement to my preoccupation with electronic games) showed the highest percentage of the level of health risks of electronic games addiction, with total weights (622), Weighted relative weight (71.74), percentage (11.16), and Ranking (1). Then the phrase (I suffer from severe pain in the shoulders and neck and inflammation in the spine due to the lack of movement while playing electronic games), with total weights (585), Weighted relative weight (67.47), percentage (10.5), and Ranking (2). Then the phrase (I developed visual impairment and eye inflammation as a result of the long time I was sitting in front of the electronic game screen), with total weights (577), Weighted relative weight (66.55), percentage (10.35), and Ranking (3). Then the phrase (I suffer from a tear in the ligaments of the joints of the hands as a result of excessive movement of my fingers on the arm of electronic games), with total weights (564), Weighted relative weight (65.05), percentage (10.12), and Ranking (4). Then the phrase (I became ill with depression as a result of the intense fear that I was exposed to during a game), with total weights (562), Weighted relative weight (64.82), percentage (10.08), and Ranking (5). The 5 types of health risks of electronic games addiction that we have reviewed are the most types of health risks that negatively affect the child, which has clearly increased after the Covid-19 pandemic, this threatens the child with great danger towards this increasing rate of health risks directed against him, which affects his normal relations with his family members. This corresponds to a study (Pontes, 2017; Khatib et al., 2018; Das et al., 2017; and Al-Bayed and Naser, 2017) which emphasized that Addiction to electronic games exposes the child to stress and poor eyesight and obesity and brain disorders, especially concentration and thinking disorders, movement of the eyes is rapid, which leads to stress and the occurrence of redness, dehydration, and dizziness due to the electromagnetic radiation emitted from the electronic screen, and headache and sometimes depression.

### 3.5 Study findings related to RQ5

The question was: Does the degree of children's awareness of their increased exposure to the risks of addiction to electronic games due to the Covid-19 pandemic vary according to gender, age, number of hours of play per day, and favorite games?

The researcher carried out an independent one-way ANOVA test to answer the fifth research question in the study to assess the significance of the differences between the averages of children's awareness of their increased exposure to the risks of addiction to electronic games due to the Covid-19 pandemic according to gender, age, number of hours of play per day, and favorite games. The results are detailed in the following section (see: Table 7):

**Table 7:** One-way ANOVA test of children responses.

		Sum of Squares	df	Mean Square	F _Statistic_	F _critical_	Sig. level
Gender	Between Groups	137	1	137	4.94	3.84∗	Significant
	Within Groups	7957	287	27.72			
	Total	8094	288				
Age	Between Groups	295	3	98.33	3.59	2.60∗	
	Within Groups	7799	285	27.36			
	Total	8094	288				
Number of hours of play per day	Between Groups	452	4	113	4.2	2.37∗	Significant
	Within Groups	7642	284	26.91			
	Total	8094	288				
Favorite electronic games	Between Groups	646	8	80.75	3.04	1.94∗	Significant
	Within Groups	7448	280	26.6			
	Total	8094	288				

∗ Statistically significant at (α 0.05).

It is clear from Table 7 that there are statistically significant differences in children's perspectives according to the variable of gender, where **F**_**Statistic**_ 4.94 is greater than **F**_**Critical**_ 3.84 at the level of statistical significance 0.05. In addition, there are statistically significant differences in children's perspectives according to the variable of age, where **F** _**Statistic**_ 3.59 is greater than **F**_**Critical**_ 2.60 at the level of statistical significance 0.05. Besides, there are statistically significant differences in children's perspectives according to the variable of number hours of play per day, where **F**_**Statistic**_ 4.2 is greater than **F**_**Critical**_ 2.37 at the level of statistical significance 0.05. Also, there are statistically significant differences in children's perspectives according to the variable of favorite games, where **F**_**Statistic**_ 3.04 is greater than **F**_**Critical**_ 1.94 at the level of statistical significance 0.05.

**Figure 1:**
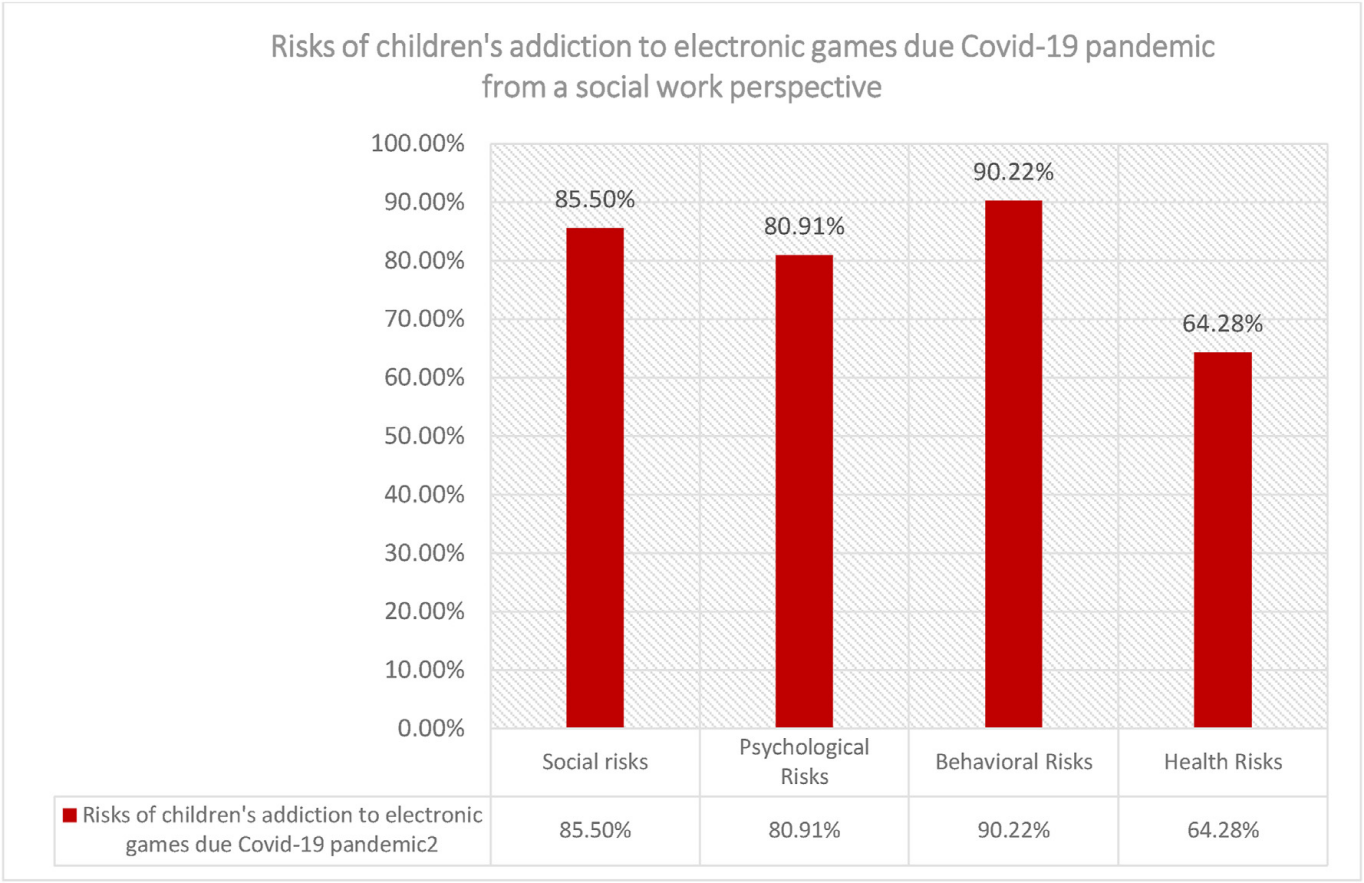
Ranking levels of the risks of children's addiction to electronic games due Covid-19 pandemic from a social work perspective.

In light of the above results that answered all the sub-questions of the study, we can now answer the main question of the study, which is {What is the impact of the Covid-19 pandemic on increasing risks of children's addiction to electronic games from a social work perspective?} (see Table 8, Figure 1).

**Table 8:** Ranking levels of the risks of children's addiction to electronic games due Covid-19 pandemic from a social work perspective.

Risks of children's addiction to electronic games due Covid-19 pandemic from a social work perspective	Total weights	Weighted relative weight of the variable	Percentage %	Level of weight representation	Ranking
Social risks	7416	85.5%	26.57	High	2
Psychological Risks	7015	80.91%	25.14	High	3
Behavioral Risks	7903	91.15 %	28.32	High	1
Health Risks	5573	64.28%	19.97	Middle	4
**Total**	**27907**		**100%**		

Weighted relative weight of the all variables in the questionnaire	80.47
Level of weight representation	High

Table 8 & Figure 1. We note the results of the answer to the main question in this study is that the value of all impacts of the Covid-19 pandemic on the increasing risks of children's addiction to electronic games came to a total weight of (27907), this is located between the High addiction level (26974–34680), So this indication is High, indicating that the level of impact is High for the Covid-19 pandemic on the increase in all types of risks of children's addiction to electronic games. It ranked first " Behavioral Risks " at 91.15%, It is followed by the ranked second “Social risks " at 85.5%, Then came third place " Psychological Risks" at 80.91%, and in finally in fourth place " Health Risks" at 64.28% which necessitates the need to take a set of serious measures to protect children from these risks.

## 4 Discussion

This study aimed to identify the impact of the Covid-19 pandemic on the increase of social, behavioral, psychological, and health risks of children's addiction to electronic games from a social work perspective. The obtained results are shown in Table 3 which showed that the impact of the Covid-19 pandemic on the increase the “Social Risks” of children's addiction to electronic games obtained a total weight of (7416), this is located between the High addiction axis level (6744–8670), So this indication is High, indicating that the level of impact is High. We have noticed that most Children's responses are indicating they became less sitting and interacting with their family members due to their great interest in electronic games at 92.73%. Also, the social relations between the children and their family members deteriorated due to their excessive interest in electronic games at 89.27%. Besides the Children tend to not attend social events of their family, colleagues, and friends because of their preoccupation with electronic games at 89.04%. in addition to the Children fell into many disputes and problems with their parents and sisters as a result of their excessive preoccupation with electronic games at 86.04%. Also, the Children became uninterested in performing their social roles due to their intense love for electronic games at 84.2%. The 5 types of social risks of electronic games addiction that we have reviewed are the most types of social risks that negatively affect the child, which has clearly increased after the Covid-19 pandemic, this threatens the child with great danger towards this increasing rate of Social risks directed against him, which affects his normal relations with his family members. This corresponds to a study (Pontes, 2017; Cha and Seo, 2018; King et al., 2019; Gong et al., 2019; and Balakrishnan and Griffiths, 2018) which emphasized Addiction to electronic games destroys the child's social development by exposing him to the risk of loses social skills in dealing with others, is unable to speak, express and tact in speaking and the child becomes shy, in addition to, his suffering from social aggression that results from accompanying bad friends.

As well, that the obtained results are shown in Table 4 which showed that the impact of the Covid-19 pandemic on the increase the “Psychological Risks” of electronic games addiction that threatens the social life of children obtained a total weight of (7015), this is located between the High addiction axis level (6744–8670), So this indication is High, indicating that the level of impact is High. We have noticed that most Children's responses are indicating they feel very frustrated when they lost in electronic games at 90.43%. Also, the Children love their imaginary world that electronic games give them more than the real world they live in at 88.35%. in addition, the Children prefer to isolate themselves from others to spend more time with electronic games at 87.77%. Besides, the Children feel very jittery and bad mood when someone forbids them from playing electronic games at 85.93%. Also, the Children see that my dependence on those around them gives them more time to play electronic games at 82.24%. The 5 types of Psychological risks of electronic games addiction that we have reviewed are the most types of Psychological risks that negatively affect the child, which has clearly increased after the Covid-19 pandemic, this threatens the child with great danger towards this increasing rate of Psychological risks directed against him, which affects his normal relations with his family members. This corresponds to a study (Saquib et al., 2017; Wang et al., 2019; Chiang et al., 2019; Hing et al., 2017; and Novrialdy et al., 2019) which emphasized that Addiction to electronic games destroys the psychological development of the child by exposing him to feelings of fear, tension, distress, excessive anxiety, depression, insomnia, and severe sadness in the child due to the terrifying and negative contents of some electronic games.

As well, that the obtained results are shown in Table 5 which showed that the impact of the Covid-19 pandemic on the increase the “Behavioral Risks” of electronic games addiction that threatens the social Life of Children obtained a total weight of (7903), this is located between the High addiction axis level (6744–8670), So this indication is High, indicating that the level of impact is High. We have noticed that most Children's responses are indicating they hit their sisters and friends in the same way that they see in electronic games at 95.73%. Also, the Children see violence against others as a manifestation of strength that they learned from electronic games at 94.81%. Besides, Children learned from their electronic games, especially in combat games, to use weapons at 94%. in addition to, the children liks with the content of electronic games that encourage abuse of others is justified in some situations at 93.19%. Also, the Children mimic the behaviors and actions they see in the electronic game at 91.81%. The 5 types of behavioral risks of electronic games addiction that we have reviewed are the most types of behavioral risks that negatively affect the child, which has clearly increased after the Covid-19 pandemic, this threatens the child with great danger towards this increasing rate of behavioral risks directed against him, which affects his normal relations with his family members. This corresponds to a study (Karlsson et al., 2019; Männikkö et al., 2020; and Nogueira et al., 2019) which emphasized that addiction to electronic games leads to the emergence of aggressive behaviors such as violence and bullying in children, so they have what is called physiological agitation that leads to automatic violence, rebellion, lethargy, and laziness, not hearing instructions and directions from their parents.

As well, that the obtained results are shown in Table 6 which showed that the Covid-19 pandemic on the increase the “Health Risks” of Electronic Games Addiction that Threatens the Social Life of Children obtained a total weight of (5573), this is located between the Middle addiction axis level (4817–6743), So this indication is Middle, indicating that the level of impact is Middle. We have noticed that most Children responses are indicating their weight increased significantly, and they became obese due to their lack of movement to their preoccupation with electronic games at 71.74%. Also, Children suffer from severe pain in the shoulders and neck and inflammation in the spine due to the lack of movement while playing electronic games at 67.47%. Besides, the Children developed visual impairment and eye inflammation as a result of the long time they were sitting in front of the electronic game screen at 66.55%. in addition to, the Children suffer from a tear in the ligaments of the joints of the hands as a result of excessive movement of their fingers on the arm of electronic games at 65.05%. Also, the Children became ill with depression as a result of the intense fear that they were exposed to during a game at 64.82%. The 5 types of health risks of electronic games addiction that we have reviewed are the most types of health risks that negatively affect the child, which has clearly increased after the Covid-19 pandemic, this threatens the child with great danger towards this increasing rate of health risks directed against him, which affects his normal relations with his family members. This corresponds to a study (Pontes, 2017; Khatib et al., 2018; Das et al., 2017; and Al-Bayed and Naser, 2017) which emphasized that Addiction to electronic games exposes the child to stress and poor eyesight and obesity and brain disorders, especially concentration and thinking disorders, movement of the eyes is rapid, which leads to stress and the occurrence of redness, dehydration, and dizziness due to the electromagnetic radiation emitted from the electronic screen, and headache and sometimes depression.

On the other hand, the obtained results, as shown in Table 7 the results indicated that the statistically significant differences in the degree of awareness of the children of increasing risks of social, psychological, behavioral and healthy to electronic games addiction due Covid-19 pandemic according to gender, age, number of hours of play per day, and type of favorite games.

In light of the above results that answered all the sub-questions of the study, we were able to answer the main question of the study as shown in Table 8, which was that the value of all impacts of the Covid-19 pandemic on the increasing risks of children's addiction to electronic games came to a total weight of (27907), weighted relative weight of (80.47%). This indication is High, indicating that the level of impact is High for the Covid-19 pandemic on the increase in all types of risks of children's addiction to electronic games. It ranked first " Behavioral Risks " at 91.15%, It is followed by the ranked second “Social risks " at 85.5%, Then came third place " Psychological Risks" at 80.91%, and in finally in fourth place " Health Risks" at 64.28%, which necessitates the need to take a set of serious measures to protect children from these risks.

## 5 Conclusion

It was clearly noticed that after the Covid-19 pandemic and children staying at home for long periods of time, The rates of risks (social - psychological - health - behavioral) of children's addiction to electronic games in its various forms have increased, especially violent games, which necessitates the need to take a set of serious measures that reflect the important recommendations of this study from the perspective of social work, which are as follows: The necessity of educating parents to monitor the content of electronic games played by their children, especially violent games, to prevent the child from playing electronic games with violent content that develop aggressive behaviors for them, in addition to, reduce the number of hours the child spends practicing these games, and to encourage parents to form a bridge of communication and constructive dialogue between them and their children, and that parents put controls and restrictions on their children's practice of electronic games to confront abnormal behavioral, psychological and social patterns such as aggression, violence, deception, lying, imitation, vigilance, physical stress, poor eyesight, distance from practicing religious rituals, academic delay, introversion, depression, intolerance, selfishness, sadness, isolation from society, social withdrawal and lack of forming social relationships and lack of communication with others. In addition, Fill the child's spare time in practicing sports and cultural activities by doing sports or reading. Also, Supporting the child's parental accompaniment to protect him from everything that may affect them as a result of electronic games. Besides, encouraging children to play the recommended educational electronic games aimed at developing intellectual and mental abilities. Also, Activating the role of the media in raising awareness of the negative behavioral patterns associated with playing electronic games and warning against their addiction. In addition, buying pets for children to take care of and take care of them to occupy their spare time. Besides, Discovering the talents of children and working to develop them. Also, encouraging children to participate in volunteer work commensurate with their age level. In addition, avoid buying electronic games devices for children under 6 years old. Besides, Educators and parents should take note of the most important positive and negative aspects of electronic games in order to work to enhance the positive aspects and reduce the effects of the negative aspects at school, home and outside the home. In addition, educating parents and those interested in child care about the electronic games classification system specified by the Entertainment Software Rating Board (ESRB) to introduce them to the classifications of electronic games according to ages, and the content of each game through descriptions of the characteristics of the game and the extent of its risk for consideration. Also, encouraging computer software companies to produce educational electronic games that contain the elements of attraction, excitement contained in popular electronic games. Also, establishing incentives and rewards to encourage children to engage in artistic, cultural and sports activities to stay away from electronic games as much as possible. Besides, parents should choose electronic games that are appropriate for the ages of their children, and be free from any content that disturbs their culture, religion and their physical, emotional and psychological health. In addition, Parents should set a specific time to play that does not exceed two hours per day to practice playing daily, provided that breaks are taken every 15 min, and then the child rest of the time is spent on practicing the rest of the daily activities. Also, Parents should not allow the child to play electronic games until after completing homework. In addition, Parents should not allow the child to play electronic games in the periods of eating meals daily. Finally, Convincing parents and children not to hesitate to communicate with specialists from social workers to benefit from their help for children in getting rid of the negative effects of the frequent practice of electronic games.

## Declarations

### Author contribution statement

Walaa Elsayed: Conceived and designed the experiments; ​Performed the experiments; ​Analyzed ​and interpreted the data; Contributed reagents, materials, analysis tools or data; Wrote the paper.

### Funding statement

This research did not receive any specific grant from funding agencies in the public, commercial, or not-for-profit sectors.

### Data availability statement

Data will be made available on request.

### Declaration of interests statement

The authors declare no conflict of interest.

### Additional information

No additional information is available for this paper.
